# Reduction of Feedback Availability Limits Self-Control Effects

**DOI:** 10.3389/fspor.2022.816571

**Published:** 2022-03-29

**Authors:** Aaron D. von Lindern, Jeffrey T. Fairbrother

**Affiliations:** ^1^Department of Health Science, College of Western Idaho, Nampa, ID, United States; ^2^School of Kinesiology, Auburn University, Auburn, AL, United States

**Keywords:** self-controlled feedback, motor learning, self-controlled learning, scarcity, limited feedback availability

## Abstract

A growing body of research has demonstrated that providing learners with self-control over aspects of the learning environment facilitates the learning of a motor skill. In applied group settings, however, the provision of feedback is at times constrained by factors such as instructor availability. The purpose of the present study, therefore, was to examine how learners ostensibly provided self-control over feedback responded when the actual availability of feedback was constrained by a predetermined schedule of a virtual coach's availability to provide feedback. Participants were divided into four feedback groups and completed 72 practice trials of a sequential key-pressing task, with three different goal movement times (900, 1,200, 1,500 ms). The KR100 group received knowledge of results (KR) after every practice trial. The KR50 group received KR on an evenly distributed quasi-randomly determined schedule after 50% of the trials. The SC group had the opportunity to request KR after every trial, but KR was only available for 50% of practice trials according to the same schedule used for the KR50 group. The YK (i.e., yoked) group received KR according to the schedule of KR received by counterparts in the SC group. Approximately 24 h after acquisition, each participant returned to complete retention and transfer tests. The retention test consisted of 15 no-KR trials of the acquisition tasks (five trials for each goal time−900, 1,200, 1,500 ms). The transfer test consisted of 15 no-KR trials with new time goals (1,300, 1,600, 1,900 ms). Results revealed a significantly lower absolute constant error (ACE) score for the SC group during transfer (*p* < 0.05), suggesting that SC effects can occur in a reduced feedback availability environment. Other measures, however, failed to show significant advantages for the SC group during any phase of the study suggesting that effects were not as robust as previous research has indicated. The results also provided some indication that perceived scarcity might have played a role in elevating the number of feedback requests in response to the reduced autonomy environment.

## Introduction

Identifying and understanding the factors that facilitate motor learning is a keystone goal in motor behavior research. Traditionally, the experimenter has determined all aspects of instructional settings in motor learning research (e.g., Nicholson and Schmidt, 1991; Schmidt, 1991; Yao et al., 1994). A growing body of research, however, points to the potential value of allowing learners to have some autonomy in shaping their experience (for reviews see Wulf, 2007; Fairbrother, 2019). Studies of so-called *self-control effects* have demonstrated that allowing learners to control some aspect of the instructional setting (e.g., the administration of feedback) facilitates motor learning compared to conditions that are controlled entirely by the researcher. Self-control experiments most often include acquisition, retention, and transfer phases. Typically, the latter two phases occur after a delay (usually 24 h). The basic experimental design compares two groups. One group is the self-control group and the other is a *yoked* control group whose schedule of instructional assistance (e.g., a feedback schedule) is created by matching each participant to a self-control counterpart. The yoking procedure originated from studies examining augmented feedback effects (e.g., Janelle et al., 1997) to ensure equivalent feedback frequencies across groups—a variable known to affect motor learning—but has been widely adopted even in studies examining other forms of instructional assistance (e.g., physical guidance or demonstrations).

Self-control effects have been found to facilitate motor learning using a number of different modes of instructional assistance, including video modeling (e.g., Wulf et al., 2005; Ste-Marie et al., 2013; Post et al., 2016), physical guidance (e.g., Wulf and Toole, 1999; Wulf et al., 2001; Chiviacowsky et al., 2012), practice schedule (e.g., Keetch and Lee, 2007; Wu and Magill, 2011), amount of practice (e.g., Post et al., 2011, 2014; Aiken et al., 2019) and augmented feedback (e.g., Janelle et al., 1995; Chiviacowsky and Wulf, 2002; Huet et al., 2009; Patterson and Carter, 2010; Aiken et al., 2012; Fairbrother et al., 2012; Lim et al., 2015; Couvillion et al., 2020). The most used manipulation of instructional assistance has been self-control over augmented feedback.

Different explanations have been forwarded to account for the effects of self-control manipulations on motor learning. One of these explanations (e.g., Chiviacowsky and Wulf, 2002) argues that the self-control allows the learner to tailor their own feedback schedule to more optimally meet their learning needs and preferences. Such tailoring may lead to more effective learning strategies compared to externally controlled schedules (Chen et al., 2002; Sanli et al., 2013). Another explanation (Janelle et al., 1997) notes that self-control may lead to deeper information processing or greater task engagement. Some studies have indicated that self-control was associated with longer preparation times (Post et al., 2011), more references to instructional materials (Aiken et al., 2012), or enhanced error detection capabilities (Chiviacowsky and Wulf, 2005; Carter et al., 2014). A third explanation posits that the provision of self-control increases the learner's motivation, self-efficacy, autonomy, and perceived competence (e.g., Chiviacowsky et al., 2012; Chiviacowsky, 2014; Wulf et al., 2014; Grand et al., 2015; Chiviacowsky and Lessa, 2017). Expanding on this is the OPTIMAL theory presented by Wulf and Lewthwaite (2016) which suggested that motivational and attentional factors combine to contribute to learning and performance by strengthening the coupling of performers' goals to their movement actions. The authors have argued that enhanced expectancies result in positive preparatory effects and that feedback delivered after so-called *good trials* potentially creates dopaminergic reinforcement cycles. This argument, however, neglects the findings showing that self-control participants do not always show preferences for feedback that confirms success (i.e., after good trials) over feedback that identifies errors (e.g., Aiken et al., 2012; Fairbrother, 2019).

Existing literature has consistently focused on establishing the self-control effect and explaining the possible theoretical mechanisms driving this effect, but has been fairly limited in the real-world applicability aspect of self-control. Considerations of real-world constraints have been limited within this research area, which presents problems for understanding the applicability of self-control findings. Of particular interest for the current study is the fact that existing research has mostly allowed self-control participants to request feedback at any time throughout the entirety of practice. When self-control has been constrained, it has been constrained in a way that has created either a partial self-control/partial prescribed condition (Patterson et al., 2011; Andrieux et al., 2015) or a condition in which the self-control was limited to a number of requests known ahead of time (Chiviacowsky and Wulf, 2005; Barrios et al., 2019). No study has considered the logical observation that real-world practice settings are often constrained by a limitation of unpredictable coach availability during practice. For example, swimmers at one end of the pool cannot receive feedback from a coach who has walked to the other end to work with another athlete and cannot predict when the coach will be back around to offer feedback opportunities.

The purpose of the present study, therefore, was to examine how learners provided limited self-control over feedback behaved when the availability of feedback was constrained by a predetermined schedule of virtual coach availability. More specifically, the present study was designed to determine if the self-control effect would be observed in such a reduced autonomy environment. Based on previous research and because of the fairly robust earlier findings related to the effects of self-control feedback schedules, it was expected that the self-control group would demonstrate superior learning compared to other groups as indicated by delayed retention and transfer tests. There could however be some presumable impact to the purported mechanisms for self-control (e.g., autonomy and motivation, improving error detection, etc.) due to the mismatch that is created when a person desires feedback but does not receive it due to the unavailability of the coach. Although testing those underlying causes is beyond the scope of this study, this experiment is an initial step to determine if restricted SC produces effects and is worth pursuing further.

## Methods

### Participants

Participants were 48 men and women (20 men, 28 women) at least 18 years of age (*M* = 21.31, *SD* = 2.82). All participants were naïve to the purpose of the study, had no prior experience with the experimental procedures and task, and provided voluntary informed consent. The University of Tennessee, Knoxville Institutional Review Board approved the protocol and informed consent form.

### Task and Apparatus

**Figure 1:**
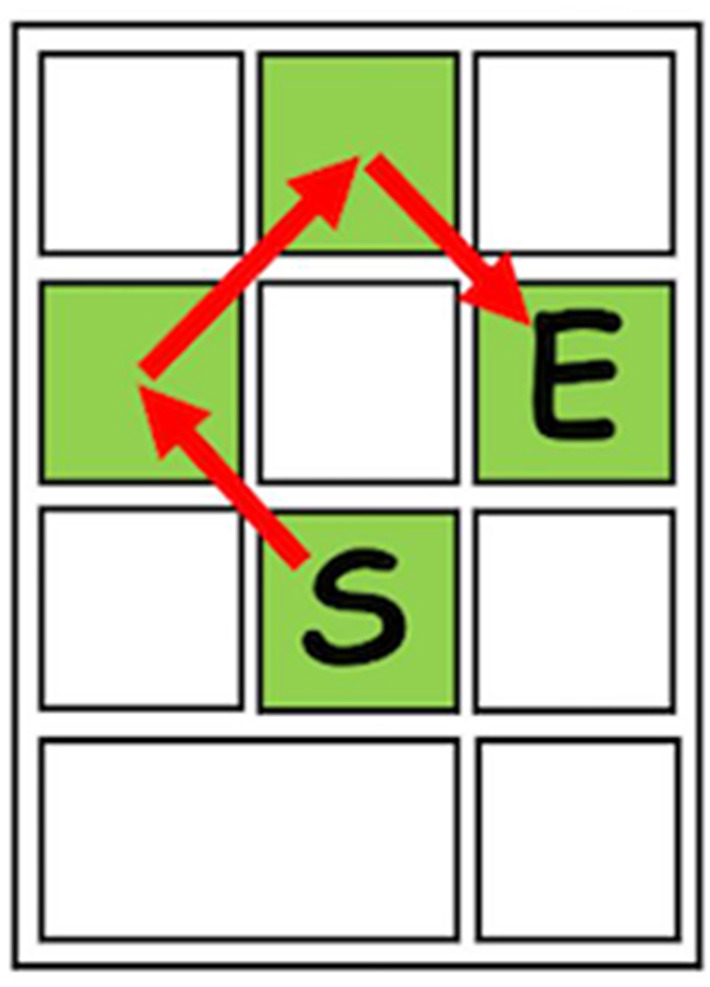
Diagram depicting the sequence of keys used in the experimental task (S, start key; E, end key).

Figure 1 depicts the sequence of keys used in the task. The experimental task was a sequential key-pressing task with movement-time goals adapted from Chiviacowsky and Wulf (2002). Participants were seated in front of a PC-compatible computer with a monitor, mouse, and keyboard (*Dell Optiplex 960*). The task required participants to press the 2, 4, 8, and 6 keys on the keyboard's numeric keypad in the order listed using the index finger of their preferred hand. For each trial during the acquisition and retention phases, participants were given overall movement time goals of 900, 1,200, or 1,500 ms, depending on the trial. During the transfer phase, the overall movement time goals were 1,300, 1,600, and 1,900 ms. The trials were presented in a predetermined and unsystematic order so it would appear to the participant that trials were randomly presented. A customized software routine written in E-Prime 2.0 (Psychology Software Tools, Inc., Pittsburgh, PA) controlled the presentation of stimuli, collection of data, and presentation of feedback.

### Procedure

Upon arrival at the laboratory, participants were welcomed and then provided voluntary informed consent. They were then quasi-randomly assigned to one of four groups. The four groups consisted of a self-control group (SC) (*n* = 12), a yoked group (YK) (*n* = 12), a 100% feedback group (KR100) (*n* = 12), and a 50% feedback group (KR50) (*n* = 12). A quasi-random assignment was used due to the nature of the SC-YK relationship where the YK participant's schedule was dependent on the schedule created by their SC counterpart. The KR100 group received knowledge of results (KR) after every trial during acquisition. The KR50 group received KR on an evenly distributed, quasi-randomly determined schedule after 50% of the trials. The SC group was instructed to only request KR when they needed it to learn the task and had the opportunity to request KR after any trial during acquisition, but was only able to receive feedback on 50% of them. The KR-trials were presented according to the same schedule used for the KR50 group. When a SC participant asked for KR after a trial that was not on the predetermined schedule, they received a message that the virtual coach was unavailable to provide feedback. The YK (yoked) group received KR according to the schedule of requests made by counterparts in the SC group. Following group assignment, participants were seated in front of the apparatus and the experimenter explained the task and procedures. To contextualize the use of a virtual coach, participants were told at the beginning of the study that the coach would provide feedback, but that he might not always be available because he was also working with other learners. This element of the procedures simulated the commonly occurring real-world constraint of reduced coach/instructor availability for one-on-one interactions within group learning settings.

Each participant completed 72 practice trials during acquisition, consisting of 24 trials for each goal time (900, 1,200, 1,500 ms). Acquisition was divided into six blocks consisting of 12 trials per block, and each block contained four trials of each goal time presented in a varied manner. Participants were instructed to begin each trial by placing the index finger of their preferred hand on the Start (S) key without depressing it. Once the goal movement time stimulus was presented, they depressed the S key and moved through the entire key sequence on a continuous motion. Participants were instructed to not begin their movement until they were prepared to complete the entire sequence without stopping. Movement Time (MT) was recorded from the moment the S key was depressed until the End (E) key was depressed. At the conclusion of each trial, participants in the SC group were given the opportunity to request KR. KR was given if the coach was available and KR was requested. KR was not given if the coach was unavailable or if KR was not requested. Participants in the other three groups were either given KR or not depending upon the associated predetermined schedule. On KR trials, the virtual coach appeared on screen and presented the participants with their MT for the trial. On no-KR trials, a screen was presented instructing participants to advance to the next practice trial, as the coach was not available to give feedback. Participants were unaware which trials would be KR trials ahead of time and KR was presented until the participant hit a button to continue to the next screen. Inter-trial intervals were controlled for on non-KR trials with a primer screen which directed participants to hit a button to move on to the next trial. Approximately 24 h after acquisition, participants returned to the laboratory to complete tests of retention and transfer. The retention test consisted of 15 no-KR trials of the acquisition tasks (five trials for each goal time−900, 1,200, 1,500 ms). The transfer test consisted of 15 no-KR trials with new time goals (1,300, 1,600, 1,900 ms).

### Data Treatment and Analysis

The primary dependent variable was movement time (MT), from which constant error (CE), absolute constant error (ACE), and variable error (VE) were calculated. MT was defined as the time between the depression of the S and E keys. MT data were collected using a customized program written within the E-prime 2.0 software package (*Psychological Software Tools, Inc*.) and raw data were exported as a CSV file for further processing using a custom routine written with the MATLAB software package (*The MathWorks, Inc*.). Constant Error (CE) was calculated from MT and defined as the difference between MT and the goal time for each trial (∑[(*MT*_*i*_−*T*)/*n*]; where *T* was the target time for a trial and *n* was the number of trials in the block for which the measure was calculated). CE indicated the average magnitude and direction of the difference between the participant's MT on a given trial and the goal time.

Absolute constant error (ACE) was calculated by taking the absolute value of CE for each block (|CE|). ACE indicated the magnitude or average timing error for each block, unaffected by canceling between subjects who achieved positive and negative CE scores. VE was calculated as the population standard deviation of CE for all trials in a block. VE indicated the participant's variability in timing accuracy around the mean of the block.

Performance measures for acquisition trials were grouped into six blocks of 12 trials for data analysis. Each block of 12 trials consisted of four trials for each of the goal times (two with feedback and two with no feedback) presented in a varied schedule. Prior to analyses, data were screened for outliers and influential scores, which were counted as errors. The threshold for identification as an outlier was ± 3 *SD* from the participant's own mean MT. Two participants' data were excluded because errors resulted in too much data loss (errors accounted for more than 33% of the data). One was in the KR50 group, and the other was in the SC group. The overall frequency of feedback requests for the SC group was 100%, which resulted in an identical feedback schedule for the YK and KR50 groups and so the YK and KR50 groups were combined into a single yoked group. CE, ACE, and VE were analyzed using separate 3 (group) ×6 (trial block) mixed design analyses of variance (ANOVA). For retention and transfer, each performance measure was grouped into a single block for each test and analyzed using separate univariate ANOVAs. For SC participants, the total number of feedback requests was counted for each acquisition block and used to calculate feedback request frequency. All analyses were conducted using measures calculated across an equal number of trials for all three goal times. Alpha levels were set at 0.05. *Post-hoc* analyses employing a Bonferroni correction were used whenever significance was obtained, and effect sizes were reported as partial eta-squared values (η^2^p). Additionally, any violations of sphericity were handled using the Greenhouse-Geisser *df* correction.

## Results

### Acquisition

**Figure 2:**
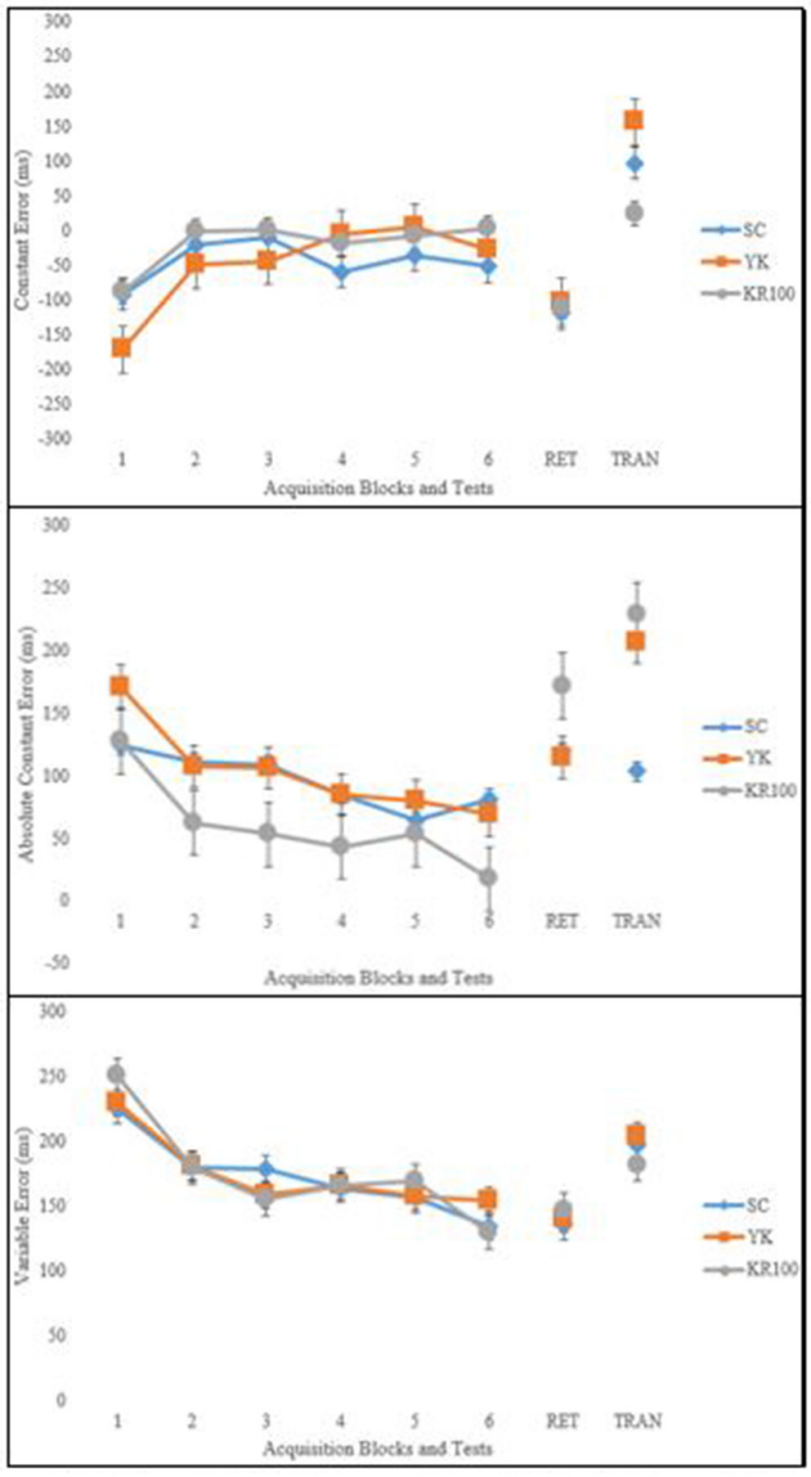
Mean (with SD error bars) CE, ACE, and VE scores for self-control (SC), yoked (YK), and 100% feedback (KR100) groups during acquisition, retention, and transfer phases (ms).

The left panels of each of the graphs shown in Figure 2 depicts the mean CE, ACE, and VE scores for the SC, KR100, and YK groups throughout each block of acquisition. All three groups showed similar mean CE scores, and all improved across acquisition blocks. These observations were supported by a significant main effect for block, *F* (5, 215) = 8.94; *p* < 0.001; η^2^*p* = 0.17. *Post-hoc* comparisons revealed that Block 1 CE was significantly larger than all other block CE scores (*p* < 0.05 for all comparisons) but that the other blocks did not differ from one another. There was no significant main effect for group, *F*_(2, 43)_ = 0.66; *p* = 0.523, nor was there a significant Group × Block interaction, *F*_(10, 215)_ = 1.69; *p* = 0.112. All three groups showed similar mean ACE scores, and all improved across acquisition blocks. These observations were supported by a significant main effect for block, *F*_(5, 215)_ = 10.93; *p* < 0.001; η^2^*p* = 0.20. *Post-hoc* comparisons revealed that Block 1 ACE was significantly larger than all other block ACE scores (*p* < 0.05 for all comparisons) but that the other blocks did not differ from one another. There was no significant main effect for group, *F*_(2, 43)_ = 2.75; *p* = 0.075, nor was there a significant Group × Block interaction, *F*_(10, 215)_ = 0.73; *p* = 0.668. All three groups showed similar mean VE scores, and all improved across acquisition blocks. These observations were supported by a significant main effect for block, *F*_(5, 215)_ = 51.66; *p* < 0.001; η^2^*p* = 0.35. *Post-hoc* comparisons revealed that Block 1 VE was significantly larger than all other block VE scores and Block 6 VE was significantly smaller than Blocks 1–3 (*p* < 0.05 for all comparisons) while all other blocks did not differ from one another. There was no significant main effect for group, *F*_(2, 43)_ = 0.01; *p* = 0.992, nor was there a significant Group × Block interaction, *F*_(10, 215)_ = 0.82; *p* = 0.577.

### Retention

The right panels of each of the graphs shown in Figure 2 depicts the mean CE, ACE, and VE scores for the SC, KR100, and YK groups during retention testing. All three groups performed similarly during retention. There were no significant effects for group observed in any of the measures, *F*_(2, 43)_ = 0.05; *p* = 0.950, for CE, *F*_(2, 43)_ = 1.11; *p* = 0.340, for ACE, and *F*_(2, 43)_ = 0.20; *p* = 0.816, for VE.

### Transfer

The right panels of each of the graphs shown in Figure 2 depicts the mean CE, ACE, and VE scores for the SC, KR100, and YK groups during transfer testing. All three groups performed similarly during transfer in terms of CE and VE. There were no significant effects for group observed for these two measures, *F*_(2, 43)_ = 1.84, *p* = 0.172, for CE and *F*_(2, 43)_ = 0.88; *p* = 0.424, for VE. For ACE, the SC group was more accurate than the YK and KR100 groups. This observation was supported a significant main effect for group, *F*_(2, 43)_ = 4.04; *p* = 0.025; η^2^*p* = 0.16. *Post-hoc* comparisons revealed that mean ACE was significantly smaller for the SC group compared to the YK and KR100 groups (*p* < 0.05 for both comparisons). In contrast, the YK and KR100 groups did not differ from one another.

## Discussion

The advantage of allowing the learner self-control over the feedback schedule has been well-established in the published literature. The OPTIMAL theory proposed by Wulf and Lewthwaite (2016) suggest that factors such as motivation and attention may be critical factors that combine to contribute to improve performance and learning. The enhanced motivational factors linked to the autonomy of allowing the learner self-control could be a possible mechanism driving the learning benefits of the self-control effect.

There remains, however, some uncertainty about the applicability of laboratory findings to practical settings that include different constraints. Current research in learner self-control of feedback has either given participants in the self-control condition the opportunity to request feedback at any time throughout the entirety of practice or created a predictable artificially constrained self-control schedule. This unrealistic scheduling of self-control creates a disconnect between laboratory findings and considerations of real-world constraints focusing on instructor or coach availability within a practice session.

The primary goal of the present study was to better understand how learners utilized control over their feedback schedule when their self-control was limited by the real-world constraint of limited coach availability during a practice session. Based on current research within the self-control paradigm, the expectation was that the self-control group would outperform the prescribed feedback groups in delayed tests providing evidence of a learning benefit for the self-control group. It was unknown, however, if limiting autonomy through a constraint on coach availability to allow feedback choices would undermine the benefits of self-control. Findings from this study indicated that that the self-control effect generalized in a limited manner to a constrained availability condition. The mixed results provided some further support for the applicability of the learning benefit of allowing self-control over augmented feedback while also indicating that a more thorough understanding of generalizability will require further research. The fact that findings differed for CE and ACE indicated that CE scores were affected by canceling caused by some participants completing the task too fast while others completed it too slow (in comparison to the timing goal). ACE scores indicated that the manipulation did produce the benefit of a lower timing error for the SC group. Additionally, the fact that significant differences appeared only in transfer and not retention are also consistent with a number of previous self-control studies in which self-control participants demonstrated better performance only during transfer testing (Chiviacowsky and Wulf, 2002; Wulf et al., 2005; Post et al., 2011; Wu and Magill, 2011; Sanli et al., 2013). Some researchers have also suggested that transfer tests could be viewed as a more sensitive means to detect learning due to the requirement of the learner to adapt a skill derived from practice to a novel task requirement (Chiviacowsky and Wulf, 2002).

A secondary result found was the lack of difference between the groups that received 50 and 100% feedback. The 100% KR group was included as a control condition to determine if reducing frequency would facilitate learning for these tasks and set of procedures. Due to the fact that the self-control group requested feedback after every trial and ended up with 50% KR, there was no frequency difference between SC, YK, and KR50. Once the KR50 and YK groups were collapsed together, there was no comparison between the KR50 and KR 100 groups. The lack of differences between the YK and KR100 groups indicated that the tasks and set of procedures were not sensitive to feedback reductions, so we can be more confident that the advantage seen was due to self-control. This finding differs from previous findings on feedback reduction.

An additional finding presented by this study related to the feedback request rate of the self-control group. Current research within self-control provides that when participants are given control over their feedback schedule, frequency of feedback requests tends to vary from relatively low (e.g., 11%; Janelle et al., 1997) to relatively higher frequencies (e.g., 35%; Chiviacowsky and Wulf, 2002; 56%; Hansen et al., 2011). Results from this study aligned more closely with the higher frequency requests and showed that within a quasi-randomized reduced-frequency schedule of opportunities to request feedback, participants in the self-control group requested feedback 100 percent of the time which is higher than we would expect based on the range of requests in previous literature. This resulted in each participant receiving feedback after 50 percent of the total trials. This elevated frequency in feedback requests may have been triggered by the uncertainty of the availability of the coach and created a sense of scarcity within feedback request chances causing participants to request feedback at every given opportunity. Although the examination of scarcity was beyond the scope of this study, it is worth noting that the high frequency of feedback requests aligned with expectations from *Commodity Theory* (Brock, 1968). Specifically, perceived value of a commodity (e.g., the opportunity to receive feedback) increases when access to that commodity becomes scarcer. Due to the random nature of the feedback request opportunities throughout practice, it may have appeared to the participants that the opportunities to receive feedback were scarce. Such a perception of scarcity may have in turn created an elevated value for the feedback and driven the observed high number of feedback requests seen in the study. Further research is needed to determine if such a perception might influence behavior in a self-controlled feedback study.

In conclusion, the findings of the present study provide more insight into the robustness of the learning benefit provided by self-control over augmented feedback. The persistence of the self-control effect within a restricted autonomy environment extends the knowledge base of the subject and helps to narrow the gap between theoretical findings and real-world constraints. It is important to note, however, that several measures did not reveal significant results, which illustrates the importance of current discussions (McKay et al., 2021; Yantha et al., 2021; Bacelar et al., 2022) about the potential benefits of self-controlled feedback for learning. Along with the observed self-control effect within a restricted environment, the elevated number of feedback requests exhibited by the self-control participants creates an interesting phenomenon that warrants further explanation into the influence of perceived scarcity on feedback requests. Future research should use a Commodity Theory perspective to explore learner behavior when feedback is perceived to be scarce and when it is perceived to be abundant.

## Data Availability Statement

Access to the raw data supporting the conclusions of this article is restricted as required by the Institutional Review Board of the University of Tennessee, Knoxville. Requests to access these datasets should be directed to the corresponding author.

## Ethics Statement

The studies involving human participants were reviewed and approved by Institutional Review Board at the University of Tennessee, Knoxville. The patients/participants provided their written informed consent to participate in this study.

## Author Contributions

AvL was the main contributor to this manuscript including the bulk of the conception and design, data collection, organization of data, statistical analysis, draft writing, and revisions. JF was a secondary contributor to this manuscript providing contributions to the statistical analysis, draft writing, and revisions. All authors contributed to the article and approved the submitted version.

## Conflict of Interest

The authors declare that the research was conducted in the absence of any commercial or financial relationships that could be construed as a potential conflict of interest.

## Publisher's Note

All claims expressed in this article are solely those of the authors and do not necessarily represent those of their affiliated organizations, or those of the publisher, the editors and the reviewers. Any product that may be evaluated in this article, or claim that may be made by its manufacturer, is not guaranteed or endorsed by the publisher.
